# Vitamin D Supplementation Improves Uterine Receptivity in a Rat Model of Vitamin D Deficiency: A Possible Role of HOXA-10/FKBP52 Axis

**DOI:** 10.3389/fphys.2021.744548

**Published:** 2021-11-25

**Authors:** Hend Ashour, Sara Mahmoud Gamal, Nermeen Bakr Sadek, Laila Ahmed Rashed, Rania Elsayed Hussein, Samaa Samir Kamar, Hayam Ateyya, Marwa Nagi Mehesen, Asmaa Mohammed ShamsEldeen

**Affiliations:** ^1^Department of Physiology, Faculty of Medicine, King Khalid University, Abha, Saudi Arabia; ^2^Department of Physiology, Kasralainy Faculty of Medicine, Cairo University, Giza, Egypt; ^3^Department of Biochemistry and Molecular Biology, Kasralainy Faculty of Medicine, Cairo University, Giza, Egypt; ^4^Department of Histology and Cell Biology, Kasralainy Faculty of Medicine, Cairo University, Giza, Egypt; ^5^Armed Forces College of Medicine, Cairo, Egypt; ^6^Department of Pharmacy Practice and Clinical Pharmacy, Faculty of Pharmacy, Future University in Egypt, Cairo, Egypt; ^7^Department of Medical Pharmacology, Faculty of Medicine, Cairo University, Giza, Egypt

**Keywords:** vitamin D, contraction, progesterone receptor, decidualization, HOXA-10/FKBP52

## Abstract

Synchronized uterine receptivity with the time of implantation is crucial for pregnancy continuity. Vitamin D (VD) deficiency has been linked to the failure of implantation. Therefore, we tested the link between the Homeobox transcription factor-10/immunophilin FK506-binding protein 52 (HOXA-10/FKBP52) axis and the uterine receptivity in VD-deficient rats. The effect of VD supplementation at different doses was also investigated. Forty-eight pregnant rats were divided into six groups (eight/group); normal control rats fed with standard chow (control), control rats supplemented with VD (equivalent dose of 400 IU/day) (control-D400). VD-deficient group (DEF) and the three VD deficiency groups with VD supplementation were equivalent to 400, 4,000, and 10,000 IU/day (DEF-D400, DEF-D4000, and DEF-D10000, respectively). The expression levels of HOXA-10/FKBP52, progesterone level, and histological evaluation of decidualization using osteopontin (OSN) and progesterone receptor (PGR) were estimated. An assessment of the uterine contractility was conducted for all rats. This study showed the downregulation of HOXA-10/FKBP52 together with increased amplitude and frequency of the uterine contractility in the DEF group compared to control. VD dose-dependent supplementation restored progesterone/receptor competency, upregulated the expressional response of HOXA-10 and its downstream FKBP52, and improved uterine receptivity and endometrial decidualization at the time of implantation that was documented by increased area% of OSN and the number of implantation beads.

## Introduction

Successful implantation is a result of complex molecular interactions between the hormonally primed uterus and mature blastocyst, which is necessary for pregnancy continuity (Ochoa-Bernal and Fazleabas, 2020). Some studies have linked vitamin D (VD) status with pregnancy loss and neonatal outcomes (Mumford et al., 2018; Fernando et al., 2020). However, the precise physiological function of VD during pregnancy is still unclear. This certainly extends far beyond the fetal calcium regulation and bone homeostasis that occurs quite late in gestation (Hewison, 2018). Gestational VD deficiency is becoming a growing concern. A recent evidence emphasized the vital role of VD during the implantation window (Von Websky et al., 2018), and its deficiency is associated with early miscarriage (Sharef et al., 2020).

Uterine receptivity is defined as the time period during which the uterus achieves proper differentiation and is ready for the initiation of implantation. In mice, the uterus becomes fully receptive on the 4th day of pregnancy (Tranguch et al., 2005). Endometrial receptivity could be assessed by several parameters, including endometrial thickness, decidualization, and the uterine blood flow (Cheng et al., 2015). Myometrial contractility regulates the extent of blood flow, if increased, it may interfere with the adequate blood supply and consequently the uterine receptivity (Cheng et al., 2015).

The active form of VD is crucial for the decidualization process, which is the key phase of implantation (Shin et al., 2011). Interestingly, both maternal decidua and fetal trophoblast express 1α-hydroxylase enzyme (CYP27B1) and are capable of producing local amounts of VD to coincide with a significant expression of vitamin D receptor (VDR) (Hou et al., 2020). Therefore, uterine tissues respond to the locally produced besides the humoral VD through the biological binding to its receptor (VDR). In their review, Ganguly et al. (2018) have emphasized the role of VD during the implantation window. The maternal decidua and fetal trophoblast expression of VD system components facilitate extravillous trophoblast invasion and establishment of early pregnancy (Ganguly et al., 2018). In line with these data, the VD level positively affects the implantation rate of the *in vitro* fertilization (Farzadi et al., 2015; Liu et al., 2019).

Homeobox transcription factor-10 (HOXA-10), a molecular multitask protein, belongs to the homeobox family of transcription factors (Guo et al., 2020) and is specifically expressed by differentiating uterine cells during the peri-implantation period (Vitiello et al., 2008) as a part of regulating the decidualization process (Ashary et al., 2020).

One of the downstream targets of HOXA-10 during implantation is the immunophilin FK506-binding protein 52 (FKBP52) (Yang et al., 2012). FKBP52 is a co-chaperone of the progesterone receptor (PGR) and is related to the uterine response to progesterone (Tranguch et al., 2007; Alaee et al., 2014). Around the implantation period, the mouse uterus demonstrates a specific pattern of FKBP52 expression, suggesting an essential role in regulating the implantation process (Daikoku et al., 2005). Meanwhile, FKBP52 protein level was decreased in the chorionic villi of recurrent spontaneous abortion patients, indicating the importance of FKBP52 in uterine receptivity in the early stages of pregnancy (Chen et al., 2015). Therefore, a HOXA-10/FKBP52 axis is thought to be a potential biomarker for predicting endometrial receptivity and blastocyst implantation (Alaee et al., 2014).

However, the link between VD and PGR signaling in the process of implantation remains unclear.

Guided by the previous data, our objectives were to investigate the role of VD in the uterine receptivity and implantation. Applying the different doses of VD was our aim to examine endometrium decidualization and myometrial contractility. Molecular determination of the cross talk between VD and PGR expression and its co-chaperone FKBP52 was investigated.

## Materials and Methods

### Experimental Animals

**FIGURE 1:**
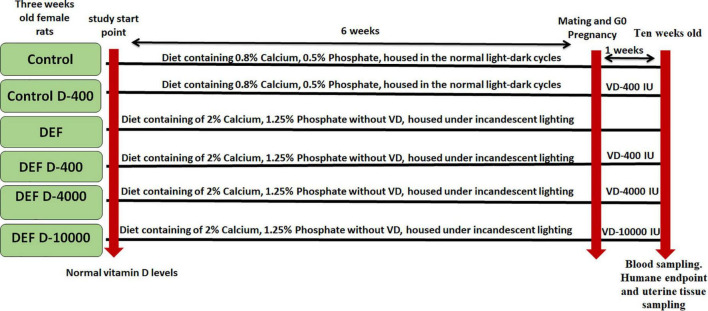
A timeline represents the included groups with the scheduled experimental interventions. D: refers to vitamin D (VD) supplementation in different doses equivalent to 400, 4,000, and 10,000 IU. DEF: refers to VD deficiency.

Forty-eight female Wistar albino rats aged about 3 weeks and weighing 60–65 g with normal plasma VD level were included in this study. All animal procedures were approved by Cairo University, Institutional Animal Care and Use Committee of Cairo University (CU-IACUC) (numbered: CU/III/F/70/17). The animals were purchased from the Animal House of the Research Institute of Ophthalmology (RIO), and the study was conducted in the RIO and Department of Physiology, Faculty of Medicine at Cairo University. The rats were housed in standard cages under room temperature (25 ± 2°C) and relative humidity (Figure 1).

### Animal Groups and Study Protocol

**FIGURE 2:**
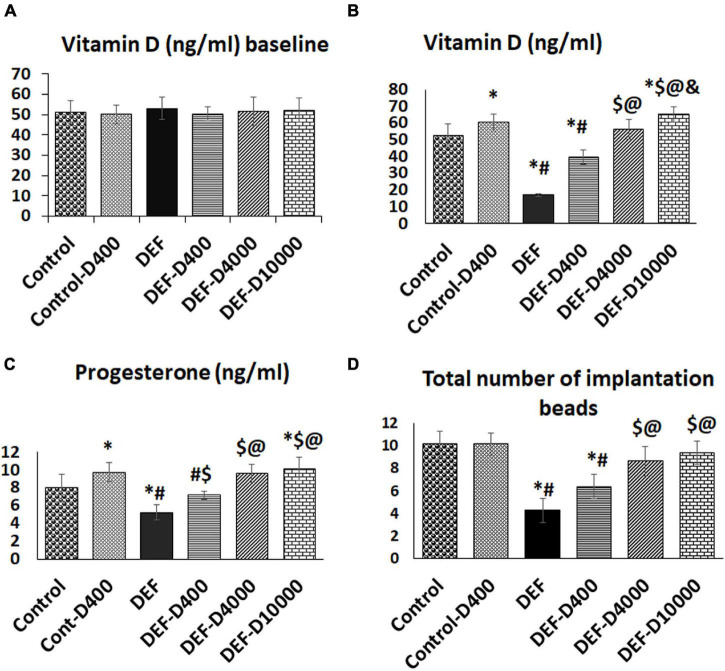
**(A)** Representation of the baseline level of VD, **(B)** the changes in the serum VD levels at the study end, **(C)** the serum progesterone, and **(D)** the number of uterine implantation sites. D: refers to VD supplementation in different doses equivalent to 400, 4,000, and 10,000 IU. DEF: refers to VD deficiency, *: A statistically significant sign compared to the control group, #: compared to control-400, $: compared to the deficiency group, @: compared to DEF-400, and &: significant compared to the DEF-4000 group (*p* < 0.05).

The rats were divided into six equal groups (eight rats each), and sample size was calculated using one-way ANOVA (G power analysis) regarding the primary outcome (the establishment of VD deficiency). VD analysis was in the normal range of the included rats at the study start point (Figure 2).

Sixteen rats were kept on the ordinary living conditions for 6 weeks to acclimatize under a light/dark cycle (light on 06:00–19:00), and then the rats were divided into: (1) Control group (*n* = 8) in which pregnancy was allowed (the method is described below). These rats were given corn oil (as a vehicle for VD at a dose of 0.5 ml/day/rat orally). Rats were followed up until the 7th day of pregnancy. (2) The control group (control-D400) with the same protocol as the control group but with VD (Euro D 10000, Euro pharm, Saint-Léonard, QC, Canada) supplementation was given in a dose equivalent to 400 IU/day starting from G0 till the time of sacrifice. The chosen dose of VD is the recommended one by the Chief Medical Officers for the United Kingdom for pregnant females (Davies et al., 2012). The dose was converted into the rat dose and calculated to be 7.2 IU/rat/day orally.

The VD deficiency model was induced in the remaining 32 female rats (as described below). Then, the pregnant rats were divided into: (3) VD deficiency group without treatment (DEF). (4) VD-deficient animals with VD supplementation of 7.2 IU/rat/day, equivalent to 400 IU (DEF-D400). (5) VD-deficient animals treated with VD in a dose of 72 IU/rat/day equivalent to human dose 4,000 IU/day (Hollis et al., 2011) (DEF-D4000). (6) VD-deficient rats treated with VD (180 IU/rat/day) equivalent to human 10,000 IU/day (Heaney et al., 2003) (DEF-D10000).

The dose conversion from human to rat was done using the Paget table (1964). VD was dissolved in corn oil, and the treatment was started from the 1st to the 7th day of pregnancy.

### Vitamin D Deficiency Model

Normal rats were kept for 6 weeks before mating on normal rat chaw diet containing 0.8% calcium, 0.5% phosphate in the normal light-dark cycles. The VD deficiency model was established by maintaining rat housing under incandescent lighting (Harms et al., 2008), to avoid VD activation by UV rays. In addition, diet administration was composed of 2% calcium, and 1.25% phosphate without VD (Stavenuiter et al., 2015). High calcium containing diet was needed to maintain normal calcium level and to prevent hyperparathyroidism (Kollenkirchen et al., 2009).

The model was proven by the low VD levels that were 16.46 ± 2.4 compared to that of control 57.59 ± 11.27 (ng/ml).

In the VD treated groups, calcium and phosphate in the diet were returned to the normal level (0.8%, 0.5%) to avoid excessive absorption and to prevent the disturbed serum levels.

### Establishment of Pregnancy

After the determination of their sexual maturity using vaginal smear, the nulliparous 9-week female rats were bred overnight with the same number of fertile males. Then, female rats were subjected to daily morning examination for the detection of the vaginal plug and the presence of spermatozoa in vaginal smear. The + ve smear determined the day 0 of gestation (G0).

### Tissue and Blood Sampling

At early morning of the 8th day of gestation, retro-orbital blood samples were withdrawn using fine heparinized catheters. Samples were collected in test tubes; plasma was separated by centrifugation and stored at −80°C until further assessment of 25 Vitamin D3 levels, progesterone, and reactive oxygen species (ROS) levels. The animals were euthanized by an intraperitoneal injection of pentobarbital (50 mg/kg i.p.) anesthesia and were sacrificed. Careful exposure of the uterine and proper dissection of both horns were made. The number of implantation sits (beads) was calculated. Then, the uterine tissues were used for biochemical analysis, assessment of contractility, and histological examination. All samples took the same codes of their included rats, and the handling of the samples and analysis was done blindly.

### Estimating Serum Levels of Total 25-Hydroxyvitamin D3 and Progesterone Level

Using ELISA, serum VD was estimated according to the manufacturer’s recommendations (Cat. No: DEIA2219, Creative Diagnostics, NY, United States), and progesterone (Cat. No: SE120087, Sigma Aldrich, St. Louis, MO, United States).

### Estimation of Serum Calcium and Phosphate Levels

Using the colorimetric assay method supplied by BioVision, Inc., CA, United States, and following the instructions of the manufacturer, the levels of calcium and phosphate were determined by the Calcium Colorimetric Assay kit (Catalog #K380-250; 250 assays) and the Phosphate Colorimetric Assay kit (Catalog #K410-500; 500 assays).

### Estimation of Uterine Hydrogen Peroxide

Uterine content of hydrogen peroxide (H_2_O_2_) levels was measured by the method of Pick (1996). Briefly, 100 μl of tissue homogenate was prepared in Tris–HCl buffer (20 mM, pH7.4), and 100 μl of the prepared assay solution was added to 10 μl of 1.0 N NaOH to get the reaction. Absorbance was recorded using the ELISA reader.

### Analysis of Uterine Glutathione Peroxidase Activity

Quantitative assessment of glutathione peroxidase (GSH-Px) activity in the uterine tissues was determined using the colorimetric Glutathione Peroxidase Assay kit (ab102530; Abcam, Cambridge, United Kingdom). The principle of the test is that GSH-Px oxidizes GSH to produce GSSG as a part of the reaction in which it reduces cumene hydroperoxide. Then, glutathione reductase reduces GSSG to produce GSH, and consumes NADPH. The reaction was measured at optical density = 340 nm.

### Quantitative Real-Time PCR of HOXA-10 and FKBP52 Expression Level

Samples from the uterine tissue were processed and homogenated, and the RNA was extracted using the SV total RNA isolation system (Promega, Madison, WI, United States). The obtained RNA was determined by spectrophotometry at 260 nm, the absorbance unit (A260) equals 40 μg of single standard RNA/ml. Then, cDNA was synthesized by using reverse transcriptase. The high capacity cDNA reverse transcription kit (#K1621, Fermentas, Waltham, MA, United States) was used for RNA conversion to cDNA. Real-time quantitative PCR (qPCR) amplification (SYBR green I) and Applied Biosystem with software version 3.1 (StepOne^®^, United States) was used for the detection and analysis of the results. A 10 μl qPCR reaction mixture was formed of the following: cDNA 1 μl, forward primer (10 μM) 0.5 μl, reverse primer (10 μM) 0.5 μl, SYBR qPCR mix 5 μl, ddH_2_O 3 μl. Cycling conditions included initial denaturation for 10 min at 95°C, then 40 cycles of denaturation for 15 s at 95°C and annealing at which the temperature was lowered to 60°C for 1 min were done till the separation of all complex targets (dsDNA) (Song and Tan, 2018). The primer sequence for FKBP52 was obtained from Sangon Biotech Co., Ltd., Shanghai, China as shown in Table 1. The accession ID of HOXA-10 is XM_008762949.1, and for the house keeping gene Beta-actin is NM_031144.3.

**TABLE 1:** Primer sequence of Homeobox transcription factor-10 (HOXA-10), FK506-binding protein 52 (FKBP52), and the housekeeping gene Beta-actin.

	Primer sequence
HOXA-10	**Sense:** 5′ AGGACTCCCTGGGCAATTC 3′
	**Antisense:** 5′ GTAAGGGCAGCGTTTCTTCC 3′
FKBP52	Sense: 5′ CACTACACTGGCTGGCTGCT 3′
	**Antisense:** 5′ TGGTTGCCACAGCAATATCC 3′
Beta actin	**Sense:** 5′ GTAGCCATCCAGGCTGTGTTG 3′
	**Antisense:** 5′ TGCCAGTGGTACGACCAGAG 3′

All cDNAs of the prepared samples (for HOXA-10 and FKBP52), internal control (housekeeping gene of Beta-actin gene expression), and non-template control (water to confirm the absence of DNA contamination in the reaction mixture) were obtained in duplicates (Table 1).

### Assessment of Uterine Contractility

The caudal halves of the uterine right horns were excised and cleaned of adhering fat and mesentery. Whole tissue strips were cut from the area in between the implantation sites into 10-mm length streps and mounted in organ baths (ADInstruments, Sydney, NSW, Australia, ML1110). The bath contained about 20 ml of Krebs’ solution buffer 130 mM NaCl, 4.7 mM KCl, 1.18 mM KH_2_PO_4_, 1.17 mM MgSO_4_, 1.16 mM CaCl_2_, 14.9 mM NaHCO_3_, and 5.5 mM dextrose (Sigma Aldrich, St. Louis, MO, United States) (Darios et al., 2012). The solution was maintained at 37°C and aerated with 95% O_2_/5% CO_2_ throughout the experimental period with washes every 15 min. The strips were initially tensioned to 1 gm and connected to an isometric force transducer (ADInstruments, Sydney, NSW, Australia, TRI210) by a silk thread. The latter was connected to a bridge amplifier (ADInstruments, Sydney, NSW, Australia, ML221), to amplify and convert the tension force generated by the contractions. The measurements were recorded by Powerlab (ADInstrument, Sydney, NSW, Australia, ML866) and analyzed by using LabChart 8 version. Preparations were allowed to equilibrate for about 60 min to obtain the spontaneous phasic contractions. After that, a continuous curve was recorded for 10 min (Aley et al., 2010).

### Histological Analysis

The pregnant uteri demonstrated scattered implantation chambers through which transverse cuts were taken at their widest area. Then, the specimens were placed in Bouin’s solution overnight, and paraffin blocks were prepared. Sections of 5-μm thickness were obtained and processed to the following histological studies:

1. H&E staining to demonstrate the structural changes in the decidual cells at the mesometrial region.
2. Immunohistochemical staining for PGRs and osteopontin (OSN), as a marker of the endometrial decidualization. Anti-PGRs monoclonal Ab (Thermo Scientific, Waltham, MA, United States, MA1-10202) and anti-OSN polyclonal antibody (Ab) (Thermo Scientific, Waltham, MA, United States, RB-9097-R7) were used. The application of the primary Abs was followed by incubation in a humidity chamber for an hour at room temperature. The sections were costained with Meyer hematoxylin to visualize the nuclei.

### Morphometric Assessment

Using the “Leica Qwin 500 C” image analyzer (Cambridge, United Kingdom), the area% of + ve PGR immunostaining in addition to the area% of + ve OSN at the mesometrial region, in 10 non-overlapping high-power fields (HPF) (×400)/rat were estimated.

### Statistical Analysis

Quantitative data were summarized as means ± SDs. Data were normally distributed (attached Supplementary File 1) and compared using the ANOVA test followed by Tukey’s *post hoc* test for multiple comparisons between the groups. Pearson’s correlation test was done to detect the relationship between the parameters among groups. The probability value < 0.05 was considered statistically significant. Calculations were made on Statistical Package of Social Science software (SPSS), version 25 (Chicago, CA, United States).

## Results

### Serum Vitamin D and Progesterone Level Changes Between Groups

The serum VD levels were significantly increased in the control group received the vitamin compared to that of control group. The induced VD deficiency model succeeded to reduce (*p* < 0.01) the serum vitamin levels compared to the control group. In the treated groups following deficiency, there was a significant (*p* < 0.05) progressive dose-dependent elevation in the serum VD level as demonstrated in Figure 2B.

Interestingly, the progesterone level followed the fluctuation in the VD level that was observed among the deficient and treated groups. Although the serum VD level was significantly higher (*p* < 0.05) in the group received 10,000 doses than the rats treated with 4,000 doses, the progesterone level showed no further elevation (data are shown in Figure 2C).

### The Total Number of Implantation Sites Was Diminished by the Model Establishment and Improved by Vitamin D Supplementation

Vitamin D-deficient rats demonstrated a significant decreased (*p* < 0.001) number of implantation sites (4.25 ± 1.03) compared to control and control-D400 groups (10.13 ± 1.1 and 10.2 ± 0.99, respectively). A progressive increase (*p* < 0.05) in the number of implantation beads was detected by VD replacement in a dose-dependent effect in the groups DEF-D400, DEF-D4000, and DEF-D10000 (6.37 ± 1.06, 8.6 ± 1.3, and 9.4 ± 1.06, respectively) (Figure 2D).

### Serum Calcium and Phosphate Levels

**FIGURE 3:**
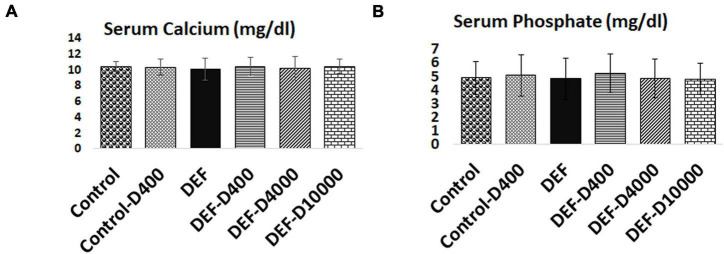
Representation of maintained serum **(A)** calcium and **(B)** phosphate within the normal range in all the groups. D: refers to VD supplementation in different doses equivalent to 400, 4,000, and 10,000 IU. DEF: refers to VD deficiency. No statistical significance between the groups.

As demonstrated in Figures 3A,B, maintained serum calcium and phosphate levels were achieved through diet adjustment to exclude their effects on the uterine contractility. There was no significant difference among groups in neither serum calcium nor phosphate.

### Oxidative Stress Markers in the Uterine Tissues in the Studied Groups

**FIGURE 4:**
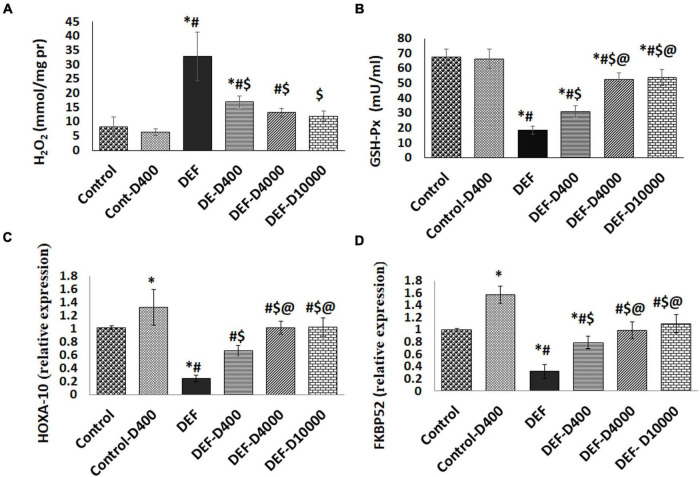
Represents the levels of **(A)** H_2_O_2_, **(B)** Glutathione peroxidase activity (GSH-Px) in uterine tissues, and the expression levels of **(C)** Homeobox transcription factor (HOXA-10), as well as **(D)** Immunophilin FK506-binding protein 52 (FKBP52). D: refers to vitamin D supplementation in different doses equivalent to 400, 4,000, and 10,000 IU. DEF: refers to vitamin D deficiency. *: Statistically significant sign compared to the control group, ^#^: compared to control-400, $: compared to deficiency group, and @: compared to DEF-400 (*P* < 0.05).

The uterine GSH-Px activity and H_2_O_2_ content were measured to trace the oxidative status in the uterine tissues. In the VD-deficient group (DEF), there was a marked (*p* < 0.001) increase in H_2_O_2_ and diminished GSH-Px activity compared to controls. In VD-deficient rats with supplementation, we detected a dose-dependent correction in both H_2_O_2_ and the GSH-Px activity data as demonstrated in Figures 4A,B. No significant change was detected between the group treated with VD 4,000 and 10,000 doses.

### Assessment of the Uterine Receptivity Marker HOXA-10

The gene expression level of HOXA-10 was significantly (*p* < 0.05) increased in the normal 400 VD dose treatment group compared to the control group. In VD-deficient rats, we reported a marked reduction (*p* < 0.001) in the HOXA-10 levels. By treating the deficient rats with VD, data showed progressive elevated HOXA-10 levels with normalization in the group supplemented with 4,000 and 10,000 VD doses (Figure 4C).

### The Modulation of Uterine Tissue Expression of FKBP52

Interestingly, the PGR co-chaperone protein FKBP52 expression levels were linked to the VD level changes. FKBP52 that was elevated (*p* < 0.05) in the normal group received 400 VD compared to the control levels (1 ± 0.02). VD deficiency resulted in a marked reduction (*p* < 0.001). FKBP52 was elevated with VD treatment in the group DEF-400 to reach the normal levels in the group treated doses equivalent to 4,000 and 10,000 (Figure 4D).

### The *in vitro* Uterine Contractility Assessment

**FIGURE 5:**
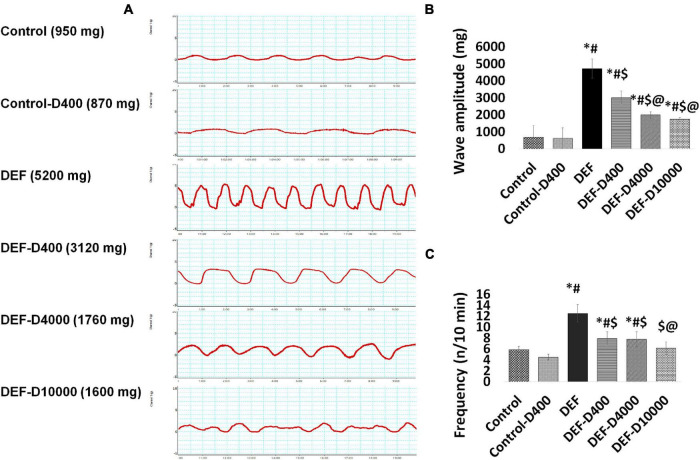
The figure represents **(A)** the curves of the uterine contraction traced during 10 min after equilibration for 60 min, the scale of the *Y* axis is 15 g and the axis is 10 min. **(B)** The amplitude (mg) and **(C)** frequency (n/10 min) of the wave were calculated. D: refers to vitamin D supplementation in different doses equivalent to 400, 4,000, and 10,000 IU. DEF: means vitamin D deficiency. *: Statistically significant sign compared to the control group, ^#^: compared to control-400, $: compared to the deficiency group, and @: compared to DEF-400 (*P* < 0.05).

Uterine contraction parameters represented by the wave amplitude and frequency of contraction in 10 min gave an idea about the uterine smooth muscle excitability status. We recorded a significant (*p* < 0.001) increase in the wave amplitude and frequency from uterine specimens obtained from the VD DEF compared to normal VD rats. In the treated groups, we noticed a significant (*p* < 0.05) progressive dose-dependent decrease in both the wave amplitude and frequency in the all administered doses compared to waves recorded from the uteri of the VD-deficient rats. The dose 10,000 did not give significant wave parameter changes from that recorded from the 4,000 dose-treated rats (Figures 5A–C).

### Morphological Assessment of the Decidualization

**FIGURE 6:**
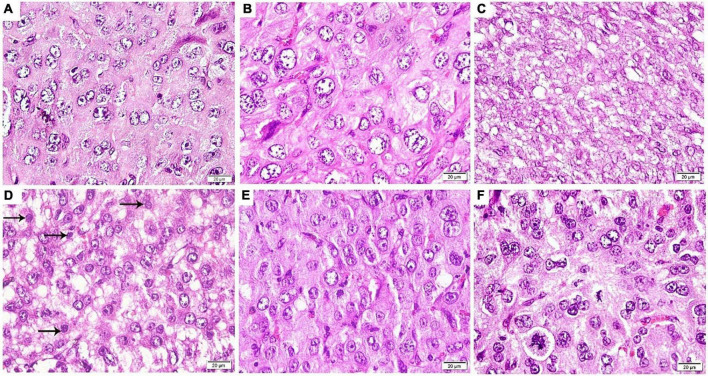
Photomicrograph of an antimesometrial region of the rat uteri at the 8th day of pregnancy (H&E ×400): **(A)** Control and **(B)** Control-D400 revealing different sized rounded decidual cells illustrating eosinophilic cytoplasm, vesicular nuclei, and numerous prominent nucleoli. **(C)** DEF displaying an impaired differentiation of stromal cells into decidual cells. **(D)** DEF-D400 illustrating alternating decidual cells and impaired differentiated stromal cells (arrows) into decidual cells. **(E)** DEF-D4000 and **(F)** DEF-D10000 revealing an obvious differentiation of stromal cells into different sized rounded decidual cells. Scale bar: 20 μm.

Histological assessment of the antimesometrial region of the rat uteri at the 8th day of pregnancy using H&E revealed proper decidualization in the control and control-D400 groups. The decidua exhibited variable-sized active decidual cells. Meanwhile, defective decidualization was detected in DEF that was evident by an impaired differentiation of stromal cells into decidual cells. The treatment with VD in DEF-D400 illustrated an impaired differentiation of some stromal cells into decidual cells. However, DEF-D4000 and DEF-D10000 displayed an obvious successful decidualization, which was comparable to the control group (Figure 6).

### Immunohistochemical Assessment of Progesterone Receptor

**FIGURE 7:**
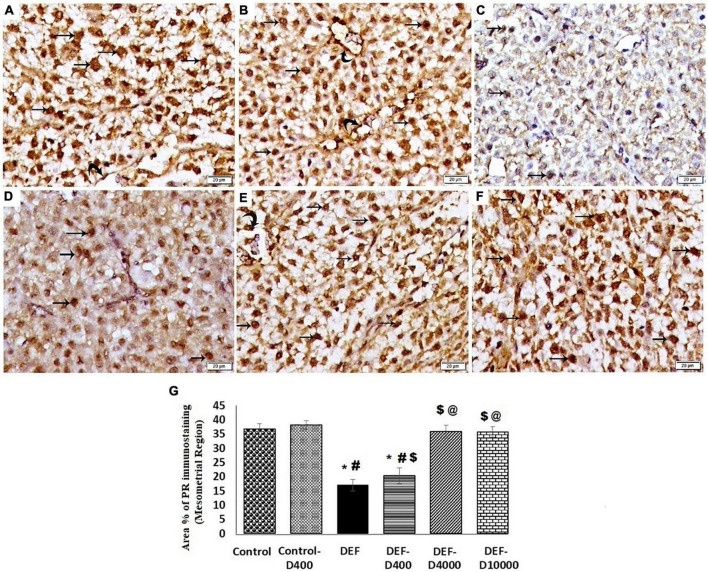
Photomicrograph of the progesterone receptor (PGR) immunostained sections in the mesometrial region of the rat uteri at 8th day of pregnancy (×400): **(A)** Control and **(B)** Control-D400 revealing strong nuclear immunostaing of PGR in the stromal cells (arrows) and vascular endothelium (curved arrows). **(C)** DEF displaying weak nuclear immunostaining of PGR in few stromal cells. **(D)** DEF-D400 illustrating moderate nuclear immunostaining of PGR in some stromal cells. **(E)** DEF-D4000 and **(F)** DEF-D10000 demonstrating obvious strong nuclear immunostaing of PGR in the stromal cells (arrows) and vascular endothelium (curved arrows). Scale bar 20 μm. **(G)** Quantification of the mean area % of PGR positive immunostaining in the mesometrial region of the rat uteri at 8th day of pregnancy. D: refers to vitamin D supplementation in different doses equivalent to 400, 4,000 and 10,000 IU. DEF: refers to vitamin D deficiency *: Statistically significant sign compared to the control group, ^#^: compared to control-400, $: compared to the deficiency group, and @: compared to DEF-400 (*P* < 0.05).

Immunohistochemical staining of PGR in the mesometrial region of the rat uteri at the 8th day of pregnancy for control and control-D400 groups displayed a strong nuclear reaction in the stromal cells and endothelial cells. This was significantly decreased in the DEF group. In DEF-D400, moderate nuclear immunostaining of PGR was detected in some stromal cells with a significant increase as compared to DEF but still significantly decreased as compared to control. DEF-D4000 and DEF-D10000 revealed substantial strong nuclear immunostaining that was comparable to the control group (Figure 7).

### Immunohistochemical Assessment of Osteopontin

**FIGURE 8:**
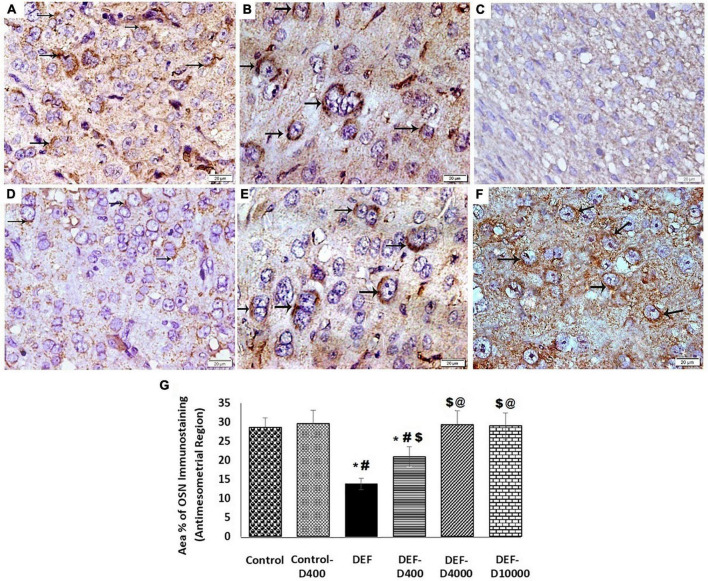
Photomicrograph of osteopontin (OSN) immunostained sections of the antimesometrial region of the rat uteri at 8th day of pregnancy (×400): **(A)** Control and **(B)** Control-D400 revealing strong cytoplasmic immunostaing of OSN in many decidual cells (arrows). **(C)** DEF displaying weak cytoplasmic immunostaining of OSN in few cells. **(D)** DEF-D400 illustrating moderate cytoplasmic immunostaining of OSN in some cells. **(E)** DEF-D4000 and **(F)** DEF-D10000 demonstrating strong cytoplasmic immunostaing of OSN in many decidual cells (arrows). Scale bar 20 μm. **(G)** Quantification of the mean area % of OSN positive immunostaining in the antimesometrial region of the rat uteri at 8th day of pregnancy. D: refers to vitamin D supplementation in different doses equivalent to 400, 4,000 and 10,000 IU. DEF: refers to vitamin D deficiency *: Statistically significant sign compared to the control group, ^#^: compared to control-400, $: compared to the deficiency group, and @: compared to DEF-400 (*P* < 0.05).

Osteopotin immunostaining of the antimesometrial region in control and control-D400 groups revealed strong cytoplasmic immunostaining of OSN in many decidual cells. In the DEF group, significant weak cytoplasmic immunostaining of OSN was detected in few cells compared to the control group. DEF-D400 illustrated a significant increase in the mean area% of OSN immunostaining compared to DEF. DEF-D4000 and DEF-D10000 groups demonstrated substantial strong cytoplasmic immunostaining of OSN in numerous decidual cells compared to the control group (Figure 8).

### Correlations

**FIGURE 9:**
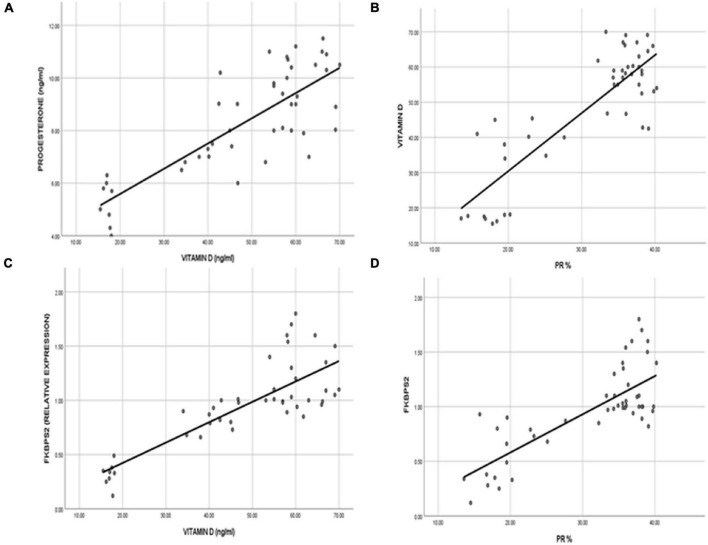
The correlation represented the direct relationship between VD levels and **(A)** the serum progesterone level and **(B)** the uterine progesterone receptor expression. VD revealed a direct correlation to the **(C)** uterine immunophilin FK506-binding protein 52 (FKBP52), which was correlated significantly to **(D)** the progesterone receptor expression in the uterine tissues.

**FIGURE 10:**
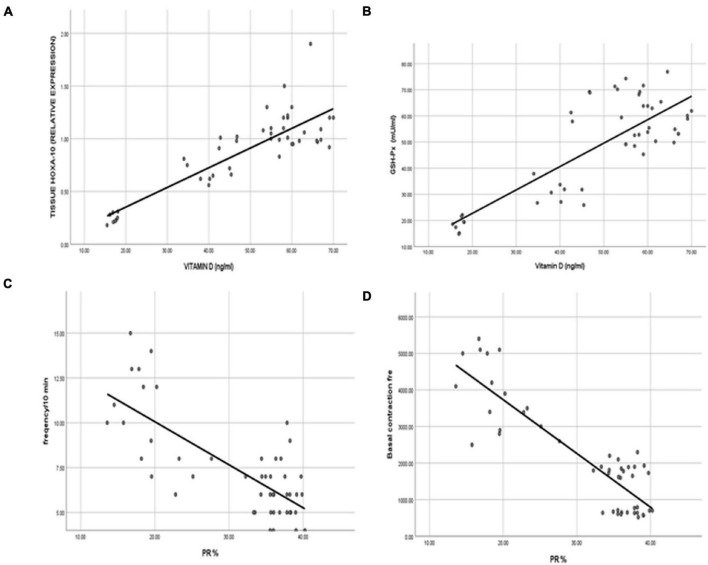
Representation of strong positive correlations between the VD levels and **(A)** HOXA-10 and **(B)** GSH-Px. Interestingly, the uterine contractility represented by **(C)** the contraction wave amplitude and **(D)** the frequency of contractions was negatively correlated to VD status.

To detect the association between the serum VD level and PGR expression and the uterine receptivity and decidualization, we revealed a strong positive correlation between the VD level and serum progesterone (*r* = 0.809), uterine PGR expression (*r* = 0.854). VD was directly correlated to the uterine PGR co-chaperone protein FKBP52 (*r* = 0.816), and the PGR expression was correlated significantly to FKBP52 (*r* = 0.778). The serum vitamin level was positively correlated to the HOXA-10 (*r* = 0.857) and to the GSH-Px levels (*r* = 0.809). However, a significant (*p* < 0.001) negative correlation was detected between the serum VD level and uterine contractility determined by the wave amplitude (*r* = − 0.877) and frequency of contraction (*r* = −0.751) (Figures 9, 10).

## Discussion

The main objectives of this work were to investigate the potential efficacy of VD supplement at different doses on endometrial decidualization and uterine receptivity as well as myometrial contractility. We monitored the tissue oxidative stress, the uterine expression of HOXA-10/FKBP52 axis, as well as the progesterone level and PGR expression in VD deficiency that was induced in rats. Here, we report that the induction of VD deficiency in female rats prior to pregnancy was associated with the decreased expression of HOXA-10/FKBP52, and PGR, a substantial augmentation of oxidative stress, and the uterine contractility. These findings were dose dependently improved by VD intake.

The proper endometrial priming with hormones is required for the successful implantation and continuation of pregnancy (Varayoud et al., 2014). Early in pregnancy, progesterone mediates an antiproliferative and anti-inflammatory role in uterine epithelium, which is being necessary for proper implantation and subsequent decidualization (Wetendorf and DeMayo, 2012). PGRs are located in the cytoplasm bound by chaperone proteins. However, upon binding to its ligand, PGRs dimerize and translocate to the nucleus, then binds to stocktickerDNA, thus, it could regulate the expression of different target genes (Wetendorf and DeMayo, 2012).

It is worth mentioning that calcitriol stimulated progesterone secretion from cultured human syncytiotrophoblasts (Barrera et al., 2007) and cumulus granulosa cells by enhancing 3β-hydroxysteroid dehydrogenase mRNA expression levels. Thereby, it could increase progesterone release (Merhi et al., 2014). In Hong et al. (2016), the authors documented decreased progesterone production from porcine ovarian granulosa cells. However, these cells were extracted from immature pigs. The relation between circulating VD levels and the outcome of pregnancy was assessed in several studies; some of them demonstrated a positive correlation between VD levels and the continuation of pregnancy. Moreover, it was reported that VD could enhance granulosa cell luteinization and increase progesterone production providing better endometrium and helping decidualization (Irani and Merhi, 2014). Herein, the current results demonstrated enhanced progesterone production *in vivo* with VD supplementation dose dependently. Therefore, VD functions on luteinization potentiation and helps decidualization. Our results showed an increased area% of PR immunostaining dose dependently in VD supplemented groups. These results were in accordance with the Hosseinirad et al.’s (2020) study, where the authors reported that VD significantly increased the expression of PGRs mRNA, protein level, and their phosphorylation in cultured endometrial stromal cells (Hosseinirad et al., 2020). FKBP52 regulates decidualization *via* promoting PGR transcriptional activity. It has been revealed that FKBP52 modulates PGR activity upon a complex formation following its binding to heat shock protein 90 (Hsp90) together with PGR (Zgajnar et al., 2019).

In FKBP52 knockout female rats, the lowered degree of decidualization was associated with a diminished progesterone response (Yang et al., 2012). Furthermore, Tranguch et al. (2007) studied the uterine changes in FKBP52 null mice prior to implantation, and found that the failure of the embryo to implant was the cause of sterility, even with normal ovulation, and was related to progesterone hormone resistance (Hirota et al., 2010). Thus, FKBP52 governs the uterine functions of PGR (Davies et al., 2005).

Many researches support the theory that the VD function in fertility is mainly implicated in an initial process of implantation (Paffoni et al., 2019). VD can modulate the expression of genes important for embryo implantation and could even interact with the local cytokines in human endometrial cells (Cermisoni et al., 2018). Abedi et al. (2019) also reported improved endometrial thickness in women subjected to a randomized trial of VD intake.

In a study conducted on human myometrial cells infected with lentivirus to stably express HOXA-10, enhanced HOXA-10 strongly suppressed the expression of contraction-associated proteins, such as connexin 43 (Cx43) and cyclooxygenase 2 (Cox2). Cx43 is responsible for cell-to-cell connections in myometrial tissues and is required for the development of synchronized myometrial contractions, whereas Cox2 catalyzes the formation of prostaglandins that could stimulate myometrial contractility following the binding to its receptors (Ramathal et al., 2010). At present, the decreased VD levels in the DEF group was associated with defective decidualization indicated by the decreased area% of OSN and a significant decrease in the mean area% of PGR; indicating the role of VD in uterine priming. All the results were reflected on a decreased number of implanted beads and FKBP52 expression levels in the DEF group as compared to the normal control rats. OSN is a member of extracellular matrix protein family implicated in cell adhesion and invasion during the implantation process (Liu et al., 2013). OSN has been shown to be expressed and upregulated 8- to 12-fold between the early and mid-secretory phase of the menstrual cycle, which is consistent with the decidualization time suggesting to be served as a marker of receptivity (Wang et al., 2018).

It was revealed that the expression and colocalization of both FKBP52 and PGR on days 4 and 5 of implantation suggesting the role of FKBP52 in decidualization as well as in modulating PGR activity during implantation (Tranguch et al., 2007).

HOXA-10 regulates the uterine levels of FKBP52, and it appears in stromal cells on gestational day 3.5 under the controlling effects of both estrogen and progesterone (Ramathal et al., 2010). HOXA-10 persists through day 4.5 when decidua starts to develop on gestational day 4.5 at the site of embryo attachment, then proliferate and differentiate extensively under controlling mechanisms of progesterone (Lim et al., 1999; Ramathal et al., 2010). The expression of HOXA-10 was found to be regulated by 1, 25(OH) D_3_ in human stromal cells. VDR and HOXA-10 protein expressions were substantially elevated in pregnant women cells compared to non-pregnant women; and VDR protein levels were positively correlated with HOXA-10 levels (Guo et al., 2020). In addition, it was documented that the intake of active VD in a dose-dependent way induced the upregulation of HOXA-10 gene expression in *in vitro* study conducted on a human endometrial stromal cell line (Du et al., 2005). HOXA10 has emerged to be a key player in both uterine development and its optimal functioning in adulthood (Ashary et al., 2020). Our results support the hypothesis that the VD-VDR system performs a role in the expression of HOXA-10. In this study, the upregulated expressional response of HOXA-10 and an improvement of the endometrial decidualization were observed to follow the intake of VD in a dose-dependent manner. An obvious successful decidualization was noted in DEF-D4000 and DEF-D10000 groups.

FKBP52 is the downstream of HOXA-10, thus the overexpressed HOXA-10 enhances FKBP52 mRNA and its protein levels (Yang et al., 2012), and eventually leading to the maintenance of PGR competency and improving its transcriptional activity (Large and DeMayo, 2012).

The present work showed that a progressive increase in HOXA-10/FKBP52 expression levels was reflected on the PGR area% and supported uterine receptivity in all the DEF groups treated with 400, 4,000, and 10,000 IU/day. However, this increase was not significant in DEF-10000 compared to the DEF-4000 group.

The potential scavenger capacity of VD was observed in the current results and evident by an increased tissue level of GSH-px in all the supplemented groups. Increased serum level of GSH-px emphasizes the major beneficial antioxidant mechanisms of VD that could support invading trophoblasts (Xu et al., 2012; Alwarfaly et al., 2014).

The oxidative stress has a negative relationship to the uterine receptivity. Hence, proper antioxidant system is essential for a successful decidualization. Decidualization is a response of maternal cells to progesterone. Human studies confirmed the role of the oxidative state and the pregnancy outcome. Under physiologic condition, progesterone enhances the activity of superoxide dismutase (SOD) in the human endometrium in early pregnancy, which in turn suppresses the production of reactive oxygen species and prostaglandin (Sugino et al., 1996). Successful implantation was correlated to a higher endometrial antioxidant; SOD, catalase (CAT) activities and the total antioxidant power (TAP), and lower lipid peroxidation (LPO) and the assessed total thiol groups (TTG) (Rahiminejad et al., 2016). Glutathione is one of the cornerstones in the intracellular ant*ioxidant system. It plays a key role in H_2_O_2_ metabolism into water and oxygen* (Rizk et al., 2013).

The regulation of oxidative stress is characterized by FKBP52. Uterine levels of the antioxidant peroxiredoxin-6 (PRDX6) were significantly diminished in the FKBP52-deficient mice leading to the implantation failure even with proper progesterone supplementation. FKBP52-deficient mice promotes H_2_O_2_-induced cell death (Hirota et al., 2010). Furthermore, induced oxidative stress downregulates HOXA-10 expression in the FKBP52-deficient mice. HOXA-10 is a crucial regulator of implantation. It mediates stromal cell proliferation and local immunosuppression (Yao et al., 2003). Thus, the low level of VD during pregnancy may enhance oxidative stress and decreases progesterone level and HOXA-10 expression (Tarcin et al., 2009). Progesterone signaling maintains uterine quiescence by suppressing the myometrial response to prostaglandin- and oxytocin-mediated inflammatory and contractile activities. Combing together, either decreasing progesterone levels or the alteration of PGR signaling may evoke uterine contractions, thus allowing the myometrium to adopt contractility (Wu and DeMayo, 2017). The results of this work revealed an increased frequency of the uterine contractility in the DEF group compared to normal one. In addition, VD supplementation could decrease the uterine contractility in a dose-dependent way. Reduced uterine peristalsis could enhance embryo implantation (Bellver and Simón, 2018). The highest prevalence of spontaneous preterm birth was detected among winter conceptions than in spring. In this context, Peng (2016) reported that VD supplementation could decrease the risk of spontaneous preterm birth by maintaining myometrial quiescence. In an *in vitro* study of human myometrial cells cocultured with monocytes, the incubation with VD decreased the expression of contractile-associated factors (Thota et al., 2014).

**FIGURE 11:**
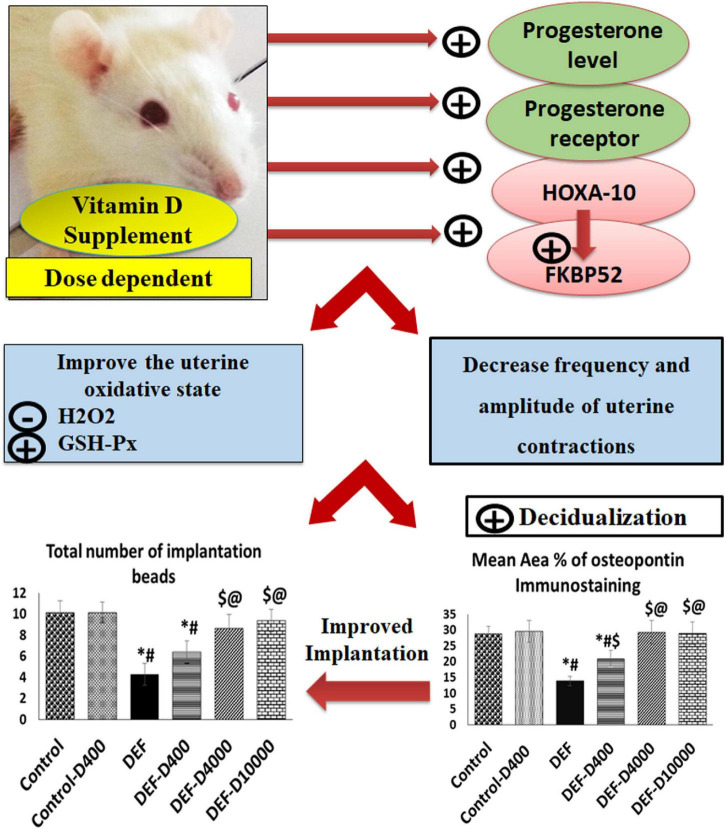
A schematic representation demonstrating the role of VD level in early pregnancy and implantation. VD-deficient rat uteri revealed a small number of uterine implantation sites (beads). By VD supplementation and in a dose-dependent effect, enhanced progesterone secretion, uterine progesterone receptor, and its co-chaperone FKBP52 expression associated with HOXA-10 upregulation were obtained. This causes improving the oxidative stress markers; GSH-px and the H_2_O_2_ levels and favors decasualization and controlled uterine contractions, thus improved uterine receptivity and promoted the implantation process. The role of VD on increased receptivity was mediated through the modulation of the immunophilin FKBP52, HOXA-10.

In conclusion, VD deficiency during the critical period of decidualization could decrease uterine receptivity and contribute to a decreased PGR level that was reflected on endometrial priming. Therefore, as demonstrated in Figure 11, an improved VD status through supplementation in the early prenatal period enhanced the expression level of HOXA-10/FKBP52, elevated immunostaining of PGR, increased glutathione activity, enhanced decidualization, and reduced the uterine contractility, and consequently improving the implantation process. As the biology of rats is consistent with that of human, VD supplementation in case of deficiency is a potential strategy for successful implantation.

### Limitations of This Study and Recommendations

Future experiments are recommended to determine the underlying mechanisms of VD-induced myometrial relaxation and ovarian progesterone secretion. An investigation of the endometrial molecular signals, which mediate the action of VD on the PGR expression and its co-chaperone, is needed. We recommend future experiments using the knockout models of HOXA-10/FKBP52 to determine decisively whether the role of VD in the regulation of PR receptors is mediated *via* the HOXA-10/FKBP52 axis or possibly the direct genomic action has an effect. Future researches are needed to employ the selected dose of VD for implementation as an efficient therapeutic strategy in human studies.

## Data Availability Statement

Data used and/or analyzed during the current study are available from the corresponding author and also found in online repositories. The names of the repository/repositories and accession number(s) can be found below: https://www.ncbi.nlm.nih.gov/genbank/, XM_008762949.1; https://www.ncbi.nlm.nih.gov/, 1; and https://www.ncbi.nlm.nih.gov/genbank/, NM_031144.3.

## Ethics Statement

The animal study was reviewed and approved by the Institutional Animal Care and Use Committee of Cairo University (CU-IACUC) (approval NO. CU/III/F/70/17).

## Author Contributions

AS and HAs: conceptualization. LR, RH and SK: methodology. LR: validation. LR, RH, HAt, MM, NS, and SK: investigation. HAs, NS, SG, and AS: data processing. AS, HAs, and SK: writing original draft preparation. SG, MM, and AS: writing, review, and editing. HAs: supervision. AS: project administration. All authors contributed to the article and approved the submitted version.

## Conflict of Interest

The authors declare that the research was conducted in the absence of any commercial or financial relationships that could be construed as a potential conflict of interest.

## Publisher’s Note

All claims expressed in this article are solely those of the authors and do not necessarily represent those of their affiliated organizations, or those of the publisher, the editors and the reviewers. Any product that may be evaluated in this article, or claim that may be made by its manufacturer, is not guaranteed or endorsed by the publisher.
