# Supplementary material for: Vitamin D Supplementation Improves Uterine Receptivity in a Rat Model of Vitamin D Deficiency: A Possible Role of HOXA-10/FKBP52 Axis
Source: Front Physiol. 2021 Nov 25;12:744548. doi: 10.3389/fphys.2021.744548 (PMC8655728; doi:10.3389/fphys.2021.744548)
Supplement: Supplementary file 1 [file Data_Sheet_1.docx]

**Journal: Frontiers in Physiology**

**Manuscript ID: 744548**

**Title: Vitamin D supplementation improves uterine receptivity in a rat model of vitamin D deficiency: possible role of HOXA-10/FKBP-52 axis**

**Authors:**

**Hend Ashour^1,2^, Sara Mahmoud Gamal^2^, Nermeen Baker^2^, Laila Ahmed Rashed^3^, Rania Elsayed Hussein^3^, Samaa Samir Kamar^4,5^, Hayam Ateyya ^6,7^,Marwa Nagi Mehesen^7^, Asmaa Mohammed ShamsEldeen*^2^**

^1^ Department of Physiology, Faculty of medicine, King Khalid University, KSA, Abha

^2^ Department of Physiology, Cairo University, Kasr Alainy, Faculty of medicine, Egypt

^3^ Department of Biochemistry and Molecular Biology, Cairo University, Kasr Alainy, Faculty of medicine, Egypt

^4^ Department of Histology and Cell Biology, Cairo University, Kasr Alainy, Faculty of medicine, Egypt

^5^ Armed Forces College of Medicine, Egypt

^6^Department of pharmacy practice and Clinical Pharmacy, Faculty of Pharmacy, Future University in Egypt, Egypt

^7^Department of Medical pharmacology, Faculty of Medicine, Cairo University, Egypt

| **Tests of Normality** | | | | | | | |
| --- | --- | --- | --- | --- | --- | --- | --- |
|  | Group | Kolmogorov-Smirnov^a^ | | | Shapiro-Wilk | | |
|  |  | Statistic | df | Sig. | Statistic | df | Sig. |
| Basal Vitamin D | Control | .197 | 8 | .200^*^ | .910 | 8 | .354 |
|  | Control-400 | .217 | 8 | .200^*^ | .915 | 8 | .392 |
|  | DEF | .238 | 8 | .200^*^ | .899 | 8 | .280 |
|  | DEF-400 | .160 | 8 | .200^*^ | .897 | 8 | .272 |
|  | DEF-4000 | .194 | 8 | .200^*^ | .934 | 8 | .555 |
|  | DEF-10000 | .160 | 8 | .200^*^ | .966 | 8 | .866 |
| Final Vitamin D | Control | .170 | 8 | .200^*^ | .969 | 8 | .887 |
|  | Control-400 | .259 | 8 | .123 | .888 | 8 | .224 |
|  | DEF | .166 | 8 | .200^*^ | .929 | 8 | .506 |
|  | DEF-400 | .144 | 8 | .200^*^ | .932 | 8 | .536 |
|  | DEF-4000 | .256 | 8 | .132 | .868 | 8 | .144 |
|  | DEF-10000 | .322 | 8 | .014 | .837 | 8 | .070 |
| Serum Progesterone | Control | .170 | 8 | .200^*^ | .957 | 8 | .779 |
|  | Control-400 | .216 | 8 | .200^*^ | .935 | 8 | .558 |
|  | DEF | .209 | 8 | .200^*^ | .939 | 8 | .599 |
|  | DEF-400 | .150 | 8 | .200^*^ | .982 | 8 | .970 |
|  | DEF-4000 | .188 | 8 | .200^*^ | .960 | 8 | .810 |
|  | DEF-10000 | .219 | 8 | .200^*^ | .910 | 8 | .354 |
| Implantation sites | Control | .216 | 8 | .200^*^ | .882 | 8 | .197 |
|  | Control-400 | .300 | 8 | .032 | .872 | 8 | .156 |
|  | DEF | .220 | 8 | .200^*^ | .917 | 8 | .408 |
|  | DEF-400 | .249 | 8 | .155 | .875 | 8 | .168 |
|  | DEF-4000 | .238 | 8 | .200^*^ | .877 | 8 | .178 |
|  | DEF-10000 | .171 | 8 | .200^*^ | .934 | 8 | .557 |
| Serum Calcium | Control | .247 | 8 | .165 | .875 | 8 | .167 |
|  | Control-400 | .138 | 8 | .200^*^ | .962 | 8 | .833 |
|  | DEF | .150 | 8 | .200^*^ | .938 | 8 | .595 |
|  | DEF-400 | .150 | 8 | .200^*^ | .908 | 8 | .338 |
|  | DEF-4000 | .251 | 8 | .147 | .900 | 8 | .289 |
|  | DEF-10000 | .143 | 8 | .200^*^ | .926 | 8 | .484 |
| Serum Phosphate | Control | .218 | 8 | .200^*^ | .853 | 8 | .103 |
|  | Control-400 | .191 | 8 | .200^*^ | .947 | 8 | .686 |
|  | DEF | .248 | 8 | .157 | .874 | 8 | .166 |
|  | DEF-400 | .250 | 8 | .150 | .927 | 8 | .488 |
|  | DEF-4000 | .179 | 8 | .200^*^ | .917 | 8 | .404 |
|  | DEF-10000 | .145 | 8 | .200^*^ | .988 | 8 | .990 |
| H2O2 | Control | .177 | 8 | .200^*^ | .930 | 8 | .520 |
|  | Control-400 | .238 | 8 | .200^*^ | .877 | 8 | .174 |
|  | DEF | .256 | 8 | .132 | .908 | 8 | .340 |
|  | DEF-400 | .208 | 8 | .200^*^ | .948 | 8 | .692 |
|  | DEF-4000 | .230 | 8 | .200^*^ | .932 | 8 | .532 |
|  | DEF-10000 | .194 | 8 | .200^*^ | .939 | 8 | .601 |
| Glutathione peroxidase | Control | .223 | 8 | .200^*^ | .946 | 8 | .666 |
|  | Control-400 | .159 | 8 | .200^*^ | .951 | 8 | .721 |
|  | DEF | .146 | 8 | .200^*^ | .937 | 8 | .583 |
|  | DEF-400 | .188 | 8 | .200^*^ | .932 | 8 | .539 |
|  | DEF-4000 | .199 | 8 | .200^*^ | .872 | 8 | .158 |
|  | DEF-10000 | .175 | 8 | .200^*^ | .954 | 8 | .752 |
| Hoxa-10 | Control | .281 | 8 | .063 | .910 | 8 | .357 |
|  | Control-400 | .287 | 8 | .050 | .811 | 8 | .038 |
|  | DEF | .148 | 8 | .200^*^ | .956 | 8 | .773 |
|  | DEF-400 | .192 | 8 | .200^*^ | .963 | 8 | .840 |
|  | DEF-4000 | .233 | 8 | .200^*^ | .908 | 8 | .338 |
|  | DEF-10000 | .227 | 8 | .200^*^ | .934 | 8 | .557 |
| FKBP-52 | Control | .315 | 8 | .019 | .871 | 8 | .155 |
|  | Control-400 | .158 | 8 | .200^*^ | .947 | 8 | .684 |
|  | DEF | .171 | 8 | .200^*^ | .961 | 8 | .815 |
|  | DEF-400 | .147 | 8 | .200^*^ | .943 | 8 | .642 |
|  | DEF-4000 | .174 | 8 | .200^*^ | .941 | 8 | .617 |
|  | DEF-10000 | .257 | 8 | .129 | .851 | 8 | .098 |
| Contraction amplitude | Control | .187 | 8 | .200^*^ | .945 | 8 | .661 |
|  | Control-400 | .137 | 8 | .200^*^ | .958 | 8 | .794 |
|  | DEF | .312 | 8 | .021 | .854 | 8 | .104 |
|  | DEF-400 | .214 | 8 | .200^*^ | .916 | 8 | .398 |
|  | DEF-4000 | .244 | 8 | .179 | .905 | 8 | .321 |
|  | DEF-10000 | .155 | 8 | .200^*^ | .941 | 8 | .623 |
| Contraction frequency | Control | .300 | 8 | .032 | .872 | 8 | .156 |
|  | Control-400 | .205 | 8 | .200^*^ | .931 | 8 | .522 |
|  | DEF | .128 | 8 | .200^*^ | .983 | 8 | .975 |
|  | DEF-400 | .210 | 8 | .200^*^ | .958 | 8 | .792 |
|  | DEF-4000 | .221 | 8 | .200^*^ | .938 | 8 | .592 |
|  | DEF-10000 | .216 | 8 | .200^*^ | .882 | 8 | .197 |
| Progesterone receptor | Control | .188 | 8 | .200^*^ | .965 | 8 | .855 |
|  | Control-400 | .137 | 8 | .200^*^ | .963 | 8 | .835 |
|  | DEF | .163 | 8 | .200^*^ | .958 | 8 | .788 |
|  | DEF-400 | .188 | 8 | .200^*^ | .974 | 8 | .927 |
|  | DEF-4000 | .121 | 8 | .200^*^ | .960 | 8 | .815 |
|  | DEF-10000 | .217 | 8 | .200^*^ | .937 | 8 | .579 |
| Osteopontin | Control | .198 | 8 | .200^*^ | .901 | 8 | .294 |
|  | Control-400 | .175 | 8 | .200^*^ | .927 | 8 | .493 |
|  | DEF | .250 | 8 | .150 | .768 | 8 | .013 |
|  | DEF-400 | .122 | 8 | .200^*^ | .972 | 8 | .910 |
|  | DEF-4000 | .166 | 8 | .200^*^ | .952 | 8 | .734 |
|  | DEF-10000 | .218 | 8 | .200^*^ | .937 | 8 | .580 |
| *. This is a lower bound of the true significance. | | | | | | | |
| a. Lilliefors Significance Correction | | | | | | | |
